# Cyberdelics: Virtual reality hallucinations modulate cognitive-affective processes

**DOI:** 10.1080/19585969.2025.2499459

**Published:** 2025-05-23

**Authors:** Giulia Brizzi, Chiara Pupillo, Clara Rastelli, Antonino Greco, Luca Bernardelli, Anna Flavia Di Natale, Silvia Francesca Maria Pizzoli, Elena Sajno, Fabio Frisone, Daniele Di Lernia, Giuseppe Riva

**Affiliations:** ^a^ Humane Technology Lab, Università Cattolica del Sacro Cuore, Milan, Italy; ^b^ Department of Psychology, Università Cattolica del Sacro Cuore, Milan, Italy; ^c^ Department of Computer Science, University of Pisa, Pisa, Italy; ^d^ Department of Psychology and Cognitive Science, University of Trento, Rovereto, Italy; ^e^ MEG Center, University of Tübingen, Tübingen, Germany; ^f^ Department of Neural Dynamics and Magnetoencephalography, Hertie Institute for Clinical Brain Research, University of Tübingen, Tübingen, Germany; ^g^ Center for Integrative Neuroscience, University of Tübingen, Tübingen, Germany; ^h^ Become-Hub, Milan, Italy; ^i^ Dep artment of Ps y chology, Università Catt olica del Sacro C uore, Milan, I taly; ^j^ Research Centre in Communication Psychology, Università Cattolica del Sacro Cuore, Milan, Italy; ^k^ Department of Pathophysiology and Transplantation, University of Milan, Milan, Italy; ^l^ Applied Technology for Neuro-Psychology Lab, IRCCS Istituto Auxologico Italiano, Milan, Italy

**Keywords:** Psychedelics, visual hallucinations, cognitive flexibility, virtual reality, artificial intelligence, DeepDream

## Abstract

**Introduction:**

Psychedelics were explored for their potential in the mental health field. However, research was delayed by concerns over short-term side effects and long-term consequences of substance use. Technological advances enabled the development of Hallucinatory Visual Virtual Experiences (HVVEs), namely psychedelic experiences simulations in immersive virtual reality. This study investigated HVVEs’ impact on cognitive flexibility, affective response, and autonomic activity.

**Methods:**

50 healthy participants underwent assessments of cognitive flexibility, control inhibition, emotional response, and autonomic activity at baseline. Participants were then exposed to two 10-minute immersive virtual reality (IVR) experiences: ‘The Secret Garden’ and its hallucinated counterpart created using Google DeepDream algorithm. All measures were presented after each video, in addition to the flow experience assessment.

**Results:**

Post-HVVE, participants demonstrated enhanced cognitive flexibility and inhibitory control. They reported increased flow-absorption and decreased flow-fluency. Both IVR experiences reduced positive affects and state anxiety compared to baseline; additionally, IVR reduced heart rate and sympathetic activity compared to baseline.

**Conclusions:**

HVVEs produced psychedelic positive effects on cognitive and emotional functioning. The complex emotional and autonomic profile mimicked awakened relaxation that, in conjunction with the cognitive flexibility enhancement, could offer the unique opportunity to exploit psychedelic advantages while mitigating risks, opening new avenues for therapeutic approaches.

## Introduction

Psychedelics have recently garnered renewed scientific interest for their therapeutic applications across several mental health conditions such as depression, anxiety, and obsessive-compulsive disorder (Anderson et al. 2020). Studies indicated that psilocybin-assisted psychotherapy led to significant improvements in depressive symptoms and increased psychological well-being (Dawood Hristova and Pérez-Jover 2023) especially in major depressive disorder and chronic treatment-resistant depression (Fuentes et al. 2019; Doss et al. 2021; Downey et al. 2023). Similarly, a recent review (Fuentes et al. 2019) highlighted that lysergic acid diethylamide (LSD) use in therapeutic settings was associated with psychiatric symptomatology reduction in addictions. Such positive effects seem to be linked to the psychedelics’ ability to induce Altered States of Consciousness (ASCs). Indeed, ASCs involve the activation of serotonin 5-HT2A receptors (Stenbæk et al. 2021). This activation is associated with increased neural plasticity, leading to alterations in cognitive processes and perceptual experiences (Shafiee et al. 2024). Research indicates that psychedelics facilitate a shift from the Default Mode Network (DMN) to more integrated and expansive states of consciousness (Carhart-Harris and Friston 2019). Neuroimaging studies showed that psychedelics induce a state characterised by increased global functional connectivity and decreased DMN activity, which correlates with subjective experiences of ego dissolution and altered perception (Yu et al. 2024). Among the theoretical frameworks proposed to explain these effects, the entropic brain hypothesis suggests that psychedelics increase the entropy of brain activity, leading to a breakdown of rigid cognitive frameworks and promoting a more anarchic state of brain function (Carhart-Harris and Friston 2019). On the cognitive side, this increased entropy is associated with heightened creativity and novel thought patterns, suggesting that psychedelics may serve as tools for enhancing Cognitive Flexibility (CF; Luppi et al. 2021).

Additionally, psychedelics were observed to modulate the autonomic nervous system, affecting Heart Rate Variability (HRV) by altering sympathetic and parasympathetic autonomic activity (Oswald et al. 2023). These physiological changes often mirror the emotional and perceptual shifts experienced during ASCs.

However, psychedelic use carries several risks—including unpredictable experiences, adverse physiological effects, and legal restrictions in many countries.

To address these challenges, technological advances proposed what are known as ‘Cyberdelics’ (Hartogsohn 2022), namely psychedelic-like experiences, generated by the combination of Immersive Virtual Reality (IVR) and deep neural networks. Cyberdelics aim to simulate the beneficial effects of psychedelics while avoiding negative effects (Suzuki et al. 2017; Aday et al. 2020; Greco et al. 2021; Rastelli et al. 2022; Magni et al. 2023). This approach relies on the Hallucination Machine (Suzuki et al. 2017) to produce Hallucinatory Visual Virtual Experiences (HVVEs), i.e., it applies deep convolutional neural networks through the Deep Dream (DD) algorithm to IVR scenario to mimic visual hallucinatory experiences (Suzuki et al. 2017; Greco et al. 2021; Suzuki et al. 2023).

Pioneering research showed that videos modified through DD improved CF, particularly inhibitory control and divergent thinking domains (Suzuki et al. 2017; Rastelli et al. 2022; Greco et al. 2024), in healthy participants. Moreover, evidence suggests that HVVEs induce neural responses akin to those seen in psychedelics. For instance, EEG studies demonstrate increased alpha and beta power following HVVEs (Mattek 2024), as well as increased entropic brain dynamics (Greco et al. 2021). Similarly, HVVEs appear to enhance global functional connectivity while disrupting rigid neural patterns, mirroring the neurodynamic reorganisation observed with psychedelics (Aday et al. 2020).

This study aimed to contribute to the emerging field of Cyberdelics by investigating how hallucinatory simulations enhance the effects of existing VR experiences. Specifically, we aimed to (i) Replicate previous findings indicating that HVVEs improve CF as assessed by divergent thinking and response inhibition. (ii) Investigate HVVEs’ impact on emotional and affective states, in particular flow experience, anxiety, distress, and negative and positive affects. (iii) Investigate HVVEs’ impact on the autonomic nervous system.

## Methods

### Participants

Fifty healthy participants (28 females, 20 males, 2 non-binary; mean age = 24.20, SD = 2.39, education (years) = 17.20, SD = 2.50) were recruited. The sample size was calculated to detect a medium effect in G*Power (*a* = 0.05, *b* = 0.80, measures = 3, *f* = 0.25, corr = 0.5). Exclusion criteria included self-reported past or current presence of neurological conditions, recurrent visual hallucinatory experiences, and the habitual use of psychoactive substances. The study was approved by the Ethics Committee of the Catholic University of Sacred Hearth of Milan (code 79-23) and all participants provided signed consent.

### Procedure

This study employed a fully within-subjects design. At baseline, participants completed sociodemographic information, and questionnaires to assess stress and anxiety levels (Subjective Units of Distress Scale (SUDS), State-Trait Anxiety Inventory (STAI-S)), affective state (Positive and Negative Affective Scale (PANAS-P, N), Self-Assessment Mannequins (SAM)), and CF tasks (Alternative Use Task (AUT) and Stroop Colour Work Task (SCW). Then, HRV was measured (2.5 min). Participants then viewed one of two IVR videos (control or HVVE). After the video presentation, HRV was measured again, along with the self-report and behavioural measures, Additionally, the Flow State Scale (FSS) was administered at this stage. This procedure was repeated for the second video. The video presentation was counterbalanced.

### Measures and instruments

#### IVR videos

**Figure 1.**
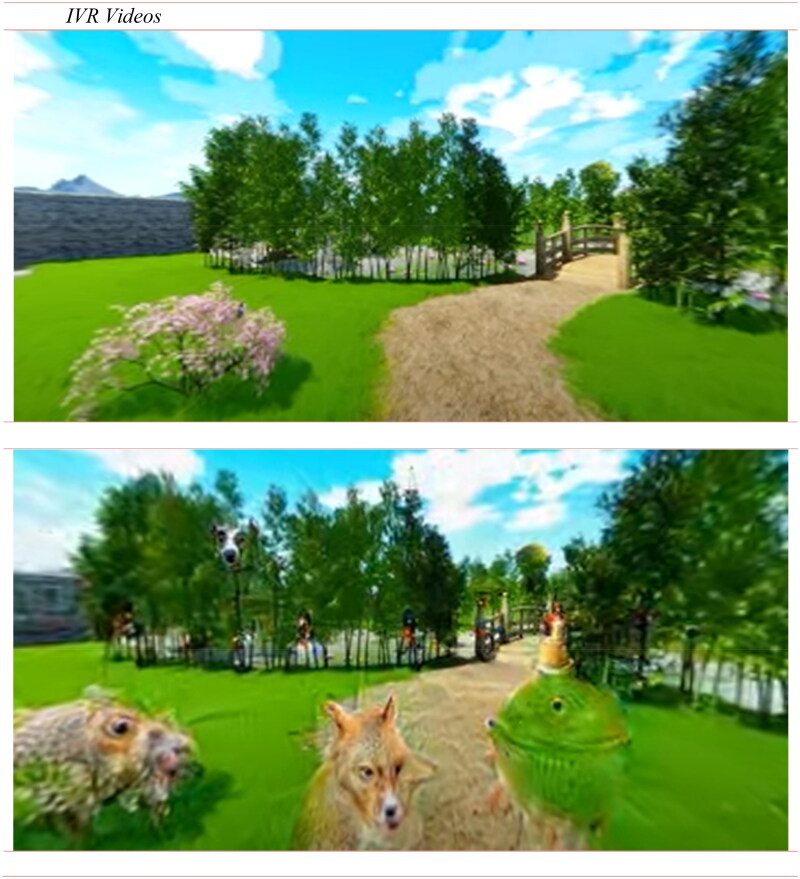
Immersive experiences were used in the current study. On the bottom panel, a screenshot of the experimental condition (HVVE condition) is presented, while on the top panel, is presented what the users saw in the control condition (original ‘The Secret Garden’).

Participants were presented with two IVR experiences. For the control condition (CC), the video ‘The Secret Garden’ was used, which is a virtual reality experience by Become Srl. It is a 10-minute 360-degree 3D video that immerses users in a Japanese garden. Previous studies revealed that it was effective in reducing work-related anxiety and stress (Riva et al. 2020; Riva et al. 2021). For the experimental condition (HVVE), the original video was modified using a DD algorithm to simulate visual hallucination (Figure 1). Details about the development procedure are provided in Supplementary Materials.

#### Measures of cognitive flexibility

The AUT is a behavioural task to measure divergent thinking (Guilford 1967; Torrance 1974). In the shortened version used in this study, participants had to list as many uses as possible for two objects (i.e., brick, pencil, paper clip, knife, cup, newspaper, shoe, ping pong ball (Palmiero and Srinivasan 2015)) in 2 min. The words’ order of presentation was randomised. Responses were scored in terms of fluency, flexibility, and originality (Vartanian et al. 2020).

The SCW (Stroop 1935) is a behavioural task assessing inhibitory control. The critical task phase involves presenting colour names where the ink colour matches the word (congruent trials) or doesn’t match the word (e.g., the word ‘red’ printed in blue ink – incongruent trials). Performance was assessed considering Stroop Task Interference Ratio (STIR), namely the difference between individual mean RT in correct incongruent trials and the mean RT in correct congruent trials divided by the mean RT in correct congruent trials (Barzykowski et al. 2021).

#### Semantic distance analysis of AUT data

We analysed AUT data exploiting recent advances in the Natural Language Processing (NLP) field, using deep language models trained on large corpora of text data. We adopted these models because of their unique ability to handle sentence-level data such as our AUT data structure. To this aim, we used the Bidirectional Encoder Representations from Transformers (BERT) model (Devlin et al. 2019), a deep neural network transformer architecture that operates with the mechanisms of attention (Vaswani et al. 2023), enabling the model to focus on various elements of the text. BERT allows us to represent both the target objects and participants’ responses as high-dimensional sentence embeddings, capturing their semantic content in a way that goes beyond surface-level similarities. We used the Italian cased BERT-XXL from HugginFace (Wolf et al. 2020), openly available at https://huggingface.co/dbmdz/bert-base-italian-xxl-uncased through the open source Python library Transformers. We passed both the target object text description and the participants’ responses as inputs to BERT, after tokenising them. To obtain a sentence embedding, we extracted the ‘CLS’ token embedding from the last hidden layer, which captures all relevant information from all the input tokens. Finally, we computed the semantic distance between each target object and associated response using the cosine distance (Rastelli et al. 2022; Rastelli et al. 2024), using the open source Python library Scipy. This metric reflects the degree of semantic divergence or similarity between the conceptual representation of the target object and the generated ideas. A higher semantic distance indicates that the participant’s response is more semantically distant or unrelated to the target object, potentially reflecting greater creative divergence or cognitive flexibility. Statistical analysis was carried out using paired one-tail t-tests to compare Baseline, HVVE, and CC conditions and false discovery rate to correct for multiple comparisons. Cohen’s d was used as a measure of effect size.

#### Measures of anxiety and affective state

The STAI-S (Pedrabissi and Santinello 1989; Spielberger 2012) is a self-report measure of state anxiety. It includes 20 items on a 4-point Likert scale (from ‘not at all’ to ‘very’). The PANAS is a questionnaire that assesses state positive (PA) and negative (NA) affects. It consists of 20 items that indicate the extent to which they feel expressed by each item, measured on a 5-point scale (from ‘not at all’ to ‘extremely’) (Watson et al. 1988; Terracciano et al. 2003). The SAM is a non-verbal tool to measure emotional dimensions associated with a stimulus, namely pleasure (valence), activation (arousal), and sense of control (dominance). Respondents must rate their emotional experience on a 5-point Likert-like scale (Bradley and Lang 1994; Di Crosta et al. 2020). The FSS (Rheinberg 2015; Rheinberg et al. 2016) is a questionnaire that assesses flow experience along four main dimensions: fluency, absorption, worry, and flow. It comprises 13 items rated on a 7-point scale (from ‘not at all’ to ‘very much’). Lastly, the SUDS (Wolpe 2018) is a visual Analogue Scale to assess the level of self-perceived distress from 0 to 100.

#### Autonomic measures

HRV was recorded using an Elite HRV’s CorSense finger pulse oximetry device (500 Hz sampling). Data was collected and processed by the KubiosHRV smartphone app. The application automatically extracts participant autonomic data and provides time-domain and frequency-domain indexes of autonomic activity indexes (Tarvainen et al. 2014). Details about autonomic measures are available in Supplementary Materials.

### Statistical analysis

Repeated measures ANOVA was conducted to analyse cognitive flexibility performances, emotional responses, and autonomic activation across the three conditions (i.e., baseline, CC, HVVE). In the case of sphericity violation, Greenhouse-Geisser correction was used. Post hoc comparisons with the Bonferroni correction were used to characterise differences between conditions. The Friedman test was used for non-normally distributed variables identified with Shapiro Test. Outliers for autonomic data were removed based on InterQuartile Range method. Natural logarithm (ln) transformations were applied to the RMSSD, HF power, and LF power autonomic indexes to normalise the data and reduce the typical variance of these measurements (Nakamura et al. 2015; Laborde et al. 2017). Sample descriptives are presented as Supplementary material. The analyses were performed using Jasp (0.18.3.0).

## Results

### Cognitive flexibility

**Figure 2.**
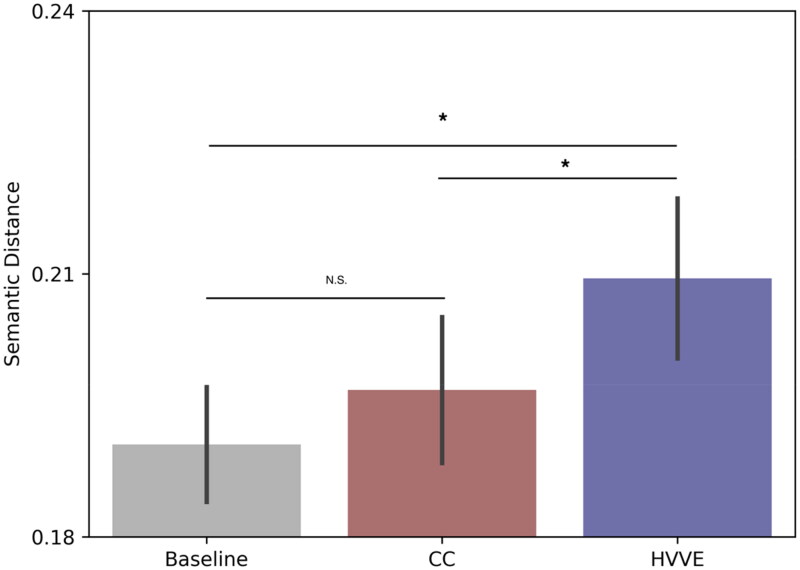
Semantic distance results from comparing sentence embeddings of target and responses of AUT. Barplots indicate average values with error bars representing standard error of the mean. Asterisks indicate statistical significance.

When comparing the sentence embeddings from the BERT model between target and response in the AUT data (Figure 2), our results revealed significant differences. The use of semantic distance as a metric allowed us to examine subtle shifts in how participants navigate semantic relationships in their responses. Specifically, semantic distance was significantly higher in the HVVE compared to the Baseline and CC condition. No difference was observed between Baseline and CC condition (see Table 2).

The statistical results for the repeated measures ANOVA and Friedman tests are summarised in Table 1, while detailed post hoc comparisons for all measures are reported in Table 2.

**Table 1.** Results of the repeated measures ANOVA and Friedman test for a within-subjects design.

	Baseline	HVVE	CC			
Measures	M(SD)	M(SD)	M(SD)	Statistics	p-value	Effect size
Cognitive flexibility						
AUT						
Flexibility	0.44 (0.12)	0.53 (0.24)	0.42 (0.14)	χ^2^(2)=13.73	<0.001***	*W* = 0.14
SCW						
STIR	0.45 (0.31)	0.31 (0.32)	0.49 (0.33)	χ^2^(2)=11.08	<0.001***	*W* = 0.11
Anxiety and affective states						
SUDS	24.40 (21.01)	29.40 (19.20)	19.40 (19.73)	χ^2^(2)=19.16	<0.001***	*W* = 0.19
STAI-S	48.52 (2.83)	38.62 (7.80)	32.92 (7.12)	F(2,98)=97.53	<0.001***	***η²_p_*** = 0.66
FSS						
Absorption		17.06 (3.97)	15.60 (4.77)	F(1,49)=5.18	0.027**	***η²_p_*** = 0.09
Fluency		25.28 (6.17)	28.42 (7.06)	F(1,49)=12.84	<0.001***	***η²_p_*** = 0.20
PANAS						
Positive	30.68 (5.30)	28.24 (7.01)	27.62 (7.26)	F(2,98)=8.92	<0.001***	***η²_p_*** = 0.15
Negative	17.42 (6.12)	14.36 (4.59)	11.86 (2.63)	χ^2^(2)=35.00	<0.001***	*W* = 0.35
SAM						
Arousal	2.90 (0.95)	3.00 (1.10)	2.32 (1.22)	F(1.76,86.06)=11.41	<0.001***	***η²_p_*** = 0.18
Autonomic measures						
HR	83.13 (12.42)	79.06 (10.92)	78.60 (10.60)	F(1.60,70.45)=11.15	<0.001***	***η²_p_*** = 0.20
SNS index	1.48 (1.44)	1.02 (1.07)	0.86 (1.03)	F(1.48,62.29)=9.94	<0.001***	***η²_p_*** = 0.19

* 0.05; **0.01; ***<0.001.

CC: control condition; HVVE: Experimental Condition; M: Mean; SD: Standard Deviation; F: F-statistic; Chi-Squared: Chi-Squared Test; Kendall’s W: Kendall’s Coefficient of Concordance. ***η*^2^_p_:** eta square.

**Table 2.** Results of post hoc comparisons for repeated measures ANOVA and Friedman test (Conover’s test).

Measures	Pairwise comparisons	Statistics for each comparison	*p*-value (adj)	Effect size (Cohen’s d)
Cognitive flexibility				
AUT				
Flexibility				
	B - HVVE	t = 2.68	0.02**	
	B – CC	t = 0.89	1	
	HVVE – CC	t = 3.57	0.002**	
Semantic distance				
	B - HVVE	t(48)=2.37	PFDR = 0.032*	d = 0.34
	B – CC	t(48)= 0.83	PFDR = 0.204	d = 0.11
	HVVE – CC	t(48)= 1.90	PFDR = 0.047*	d = 0.21
SCW				
STIR				
	B - HVVE	t = 2.60	0.03**	
	B – CC	t = 0.50	1	
	HVVE – CC	t = 3.10	0.008**	
Anxiety and affective states				
SUDS	B - HVVE	t = 1.73	0.25	
	B – CC	t = 2.63	0.030**	
	HVVE – CC	t = 4.36	<0.001***	
STAI-S	B - HVVE	MD = 9.90 [7.14, 12.65], SE = 1.13	<0.001***	1.56 [0.98, 2.14]
	B – CC	MD = 15.60 [12.84, 18.35], SE = 1.13	<0.001***	2.46 [1.72, 3.21]
	HVVE – CC	MD = 5.70 [2.94, 8.45], SE = 1.13	<0.001***	0.90 [0.41, 1.38]
FSS				
Absorption	HVVE – CC	MD = 1.46 [0.17, 2.74], SE = 0.64	0.02**	0.33 [0.03, 0.63]
Fluency	HVVE – CC	MD = −3.14 [–4.90, −1.38], SE = 0.87	<0.001***	−0.47 [–0.73, −0.19]
PANAS				
Positive	B - HVVE	MD = 2.44 [0.57, 4.30], SE = 0.76	0.006**	0.37 [0.07, 0.66]
	B – CC	MD = 3.06 [1.19, 4.92], SE = 0.76	<0.001***	0.46 [0.15, 0.77]
	HVVE – CC	MD = 0.62 [–1.24, 2.48], SE = 0.76	1	0.09 [–0.19, 0.37]
Negative	B - HVVE			
	B – CC			
	HVVE – CC			
SAM				
Arousal	B - HVVE	MD= −0.10 [-0.47, 0.27]	1	0.09 [–0.43, 0.25]
	B – CC	MD= 0.58 [0.20, 0.95]	<0.001***	0.52 [0.16, 0.89]
	HVVE – CC	MD= 0.68 [0.30, 1.05]	<0.001***	0.61 [0.24, 0.99]
Autonomic measures				
HR	B - HVVE	4.06 [1.49, 6.64]	<0.001***	0.35 [0.11, 0.60]
	B – CC	4.53 [1.95, 7.11]	<0.001***	0.40 [0.14, 0.65]
	HVVE – CC	0.46 [–2.11, 3.04]	1	0.04 [–0.18, 0.27]
SNS index	B - HVVE	0.45 [0.10, 0.80]	0.007**	0.37 [0.06, 0.68]
	B – CC	0.62 [0.27, 0.97]	<0.001***	0.51 [0.19, 0.84]
	HVVE – CC	0.16 [–0.18, 0.52]	0.73	0.14 [–0.15, 0.43]

* 0.05; **0.01; ***<0.001.

B: baseline; CC: control condition; HVVE: experimental condition; t : t-statistic for each comparison; MD: mean difference; SE: Standard error; Cohen’s d: Cohen’s d effect size; p-value (adj): Bonferroni-adjusted p-value for each comparison; [ ] = 95% confidence interval.

Regarding AUT traditional scoring, the HVVE condition led to significantly higher Flexibility compared to both the Baseline and CC conditions. Participants exposed to HVVE demonstrated enhanced cognitive flexibility relative to other conditions.

Additionally, HVVE reduced control inhibition (as measured by the STIR index) compared to both Baseline and CC conditions, indicating increased inhibitory control.

### Emotional state

HVVE elicited distress (SUDS) levels comparable to Baseline but higher than CC. For arousal (SAM), HVVE resulted in higher levels than CC, with no differences from Baseline. HVVE significantly reduced state anxiety (STAI-S) compared to Baseline but resulted in higher anxiety levels than CC. For affect (PANAS), both HVVE and CC reduced positive affect relative to Baseline, with HVVE eliciting higher negative affect compared to CC but no differences from Baseline. Regarding flow (FSS), HVVE was associated with greater absorption but reduced fluency relative to CC.

Overall, this suggests that HVVE may foster deeper immersion but doesn’t provide the calming effects and ease of engagement seen in CC.

### Autonomic measures

The HVVE condition resulted in a reduction in HR and SNS activity compared to Baseline, similar to the CC condition. Both conditions showed equivalent reductions in physiological arousal measures, suggesting a calming effect relative to Baseline.

No additional significant differences were observed for the HVVE condition across other measures. Complete analyses are provided as Supplementary Materials.

## Discussion

This study compared the impact of The Secret Garden IVR relaxing video and its hallucinated counterpart HVVEs on cognitive and affective processes. We investigated HVVEs’ effects on cognitive flexibility, affective state, and autonomic activation. The results suggest that artificially induced hallucinatory experiences were able to enhance cognitive flexibility in terms of divergent thinking and inhibitory controls in healthy participants (Rastelli et al. 2022). Results extended previous findings as the semantic analysis indicated an improvement in semantic flexibility after video exposure. We found that the semantic distance between the target object and participants’ responses to that object for alternative uses was higher after being exposed to simulated visual hallucinations compared to the control conditions. This suggests that participants were facilitated to explore the semantic space in search of a more divergent solution, in line with previous findings (Rastelli et al. 2022). Notably, we extended these previous results, which were achieved *via* cognitive network science methods, using current state-of-the-art Large Language Models for NLP analyses. Crucially, our findings seem to suggest that the negative results found in a recent study (Greco et al. 2024) about DeepDream-induced hallucinations affecting language processing could be ascribed to the different instructions given to the participant. Specifically, we prompted our participants to give a ‘creative’ response, and this resulted in a significant modulation of the language processing domain.

These effects mirror those observed with psychedelics, suggesting that simulated hallucinations can replicate the effects of these substances. Indeed, research showed that psilocybin use reduced latent inhibition and promoted more fluid associative thinking (Carhart-Harris and Friston 2019), while ayahuasca enhanced executive functions, including inhibitory control (Bouso et al. 2013).

These improvements in CF have been linked to changes in brain activity and connectivity. Specifically, psychedelic use has been observed to increase brain entropy, decrease complexity in frontal brain regions, and promote communication between generally less interactive brain networks (Carhart-Harris et al. 2014; Tagliazucchi et al. 2016; Greco et al. 2021). A better understanding of this phenomenon would open new avenues for the treatment of pathological conditions in which reduced CF plays a key role in maintaining symptomatology, such as eating disorders (Mora-Maltas et al. 2023). Similarly, it has been seen that mindfulness meditation in VR, especially open-monitoring meditation (Rebecchi and Hagège 2022), and relaxing naturalistic virtual environments (Spano et al. 2023) improve divergent thinking and CF because it requires the ability to change perspective from current cognitive perspectives. These non-drug-based approaches, like HVVEs, leverage altered states of consciousness to foster cognitive flexibility and emotional regulation. However, unlike mindfulness or relaxation-based VR therapies, HVVEs uniquely simulate hallucinatory ­experiences, potentially engaging brain networks associated with psychedelic effects, such as increased entropy and interconnectivity. Exploring these parallels and distinctions offers important insights into how HVVEs may provide a complementary or alternative approach to enhancing CF.

Regarding the emotional state, results indicated that HVVE exposure was able to enhance the level of deep absorption in the Flow subscale, despite reduced fluency. These results show that the HVVE experience was engaging and stimulating, potentially inducing states of heightened engagement like those observed with psychedelics (Studerus et al. 2012). However, the lower fluency scores indicate a possible trade-off between immersion and ease of cognitive processing. This dichotomy may reflect the challenging nature of integrating novel perceptual experiences, where increased cognitive effort is required, leading to a temporary decrease in fluency while maintaining a high absorption level. This dynamic can be explained by the visual complexity of HVVE and studies showing that flow requires effortful attention (Harris et al. 2017). Visual hallucinations of HVVE are highly intricate and dynamic, requiring the maintenance of attentional resources to interpret and integrate unfamiliar sensory input (Suzuki et al. 2023). Such visual complexity may enhance immersion by capturing attention and sustaining engagement, but it can also hinder fluency by placing greater demands on the cognitive resources required for fluid processing. Furthermore, Lavoie and colleagues (Lavoie et al. 2022) found that absorption tends to be a result of flow, related to presence and behavioural engagement, whereas fluency depends more on familiarity and skill, which are less applicable to the novel and dynamic experiences provided by HVVEs. Similarly, Carhart-Harris and colleagues (Carhart-Harris et al. 2016; Carhart-Harris and Nutt 2017) observed that psychedelics can lead to ASCs characterised by reduced connectivity in brain regions associated with self-awareness. This increases sensory uptake since there is reduced filtering of the default mode network. However, it may reduce fluency in tasks requiring sustained attention and smooth cognitive progression, as it makes it more difficult to maintain concentration and effectively integrate thoughts and actions.

Interestingly, both IVR conditions, independently from the hallucinatory manipulation, revealed a decreased positive affect (e.g., lower PANAS) and anxiety (e.g., STAI-S score). The anxiety result aligns with previous studies demonstrating the relaxing effect of The Secret Garden video (Riva et al. 2020). Then, it might be that the relaxing effect observed after IVR exposure is partially due to video content (Riva et al. 2021; Pallavicini et al. 2022). This result is further supported by a broader body of literature showing that exposure to naturalistic VR (Spano et al. 2023) environments can reduce anxiety, including among individuals with generalised anxiety disorder. If this is the case, the observed difference between the control and the HVVE condition might reflect a tendency of hallucinatory experience to contrast deep relaxation.

Another possibility that might explain HVVE’s effect on emotional state reflects the complexity and contradictory emotional richness typically associated with psychedelic experiences. Indeed, psilocybin research showed that users reported a multifaceted emotional profile linked to substance use, characterised by intense positive and negative emotional states and a feeling of deep immersion in the experience (Barrett et al. 2016). Furthermore, while mystical experiences induced by psychedelics are frequently associated with enhanced feelings of unity and connectedness, they can also elicit negative emotional responses, such as discomfort or unease. These effects may transiently diminish positive affect and can be influenced by the individual’s pre-experience emotional predisposition (Ko et al. 2022).

This ambivalence mirrors the concept of ‘pivotal mental states’ (Brouwer and Carhart-Harris 2021), namely transformative psychological states leading to significant cognitive and emotional insights and personal growth (Roseman et al. 2018). Going further, the increased absorption observed in this study aligns with the intense focus and altered sense of reality reported during psychedelic experiences. Specifically, Preller and Vollenweider (Preller et al. 2018) describe how psychedelics can induce states of deep engagement with bodily internal or external information, often accompanied by a sense of timelessness and altered self-awareness. Future research might investigate whether HVVEs might prompt self-body connection and mindfulness. This might improve interventions for conditions characterised by alterations in self-conscience (Fadiman and Korb 2019).

Additionally, our findings also highlight the ­potential role of subjective experiences in some psychedelic-induced benefits. For instance, Yaden and Griffiths (Yaden and Griffiths 2021) emphasise that subjective experiences are central to the acute effects of psychedelics, such as emotional breakthroughs and shifts in perception, which are thought to mediate their therapeutic impact. Davis and colleagues (Davis et al. 2020) further identify psychological flexibility as a critical mediator, linking the acute effects of psychedelics with reductions in depression and anxiety. This aligns with our findings, as HVVE-induced improvements in CF could similarly support psychological flexibility, which is central to adaptive emotional and cognitive functioning. However, it is important to note that subjective effects alone may not fully account for the benefits of psychedelics. Olson (Olson 2021) cautions that these effects are part of a broader set of mechanisms that include pharmacological and contextual factors. This is reflected in our results, where HVVEs, despite enhancing absorption, reduced fluency and moderated the relaxing effects of the relaxation condition.

Finally, the results of autonomic activation revealed that both IVR experiences reduced HR and SNS index compared to baseline, suggesting a general calming effect. Even though this effect might be partially related to video content, the autonomic profile seems to mimic the results of psilocybin research. A decrease in amygdala reactivity to negative stimuli has been observed in conjunction with profound emotional experiences, reflecting a dissociation between subjective intensity and physiological calming effects (Kraehenmann et al. 2015). This may reflect a state of ‘awakened relaxation’ (Amihai and Kozhevnikov 2014), where participants experience increased cognitive engagement while remaining physiologically regulated. This state could be compared to the ‘optimal arousal’ level, where a moderate level of physiological activation supports enhanced cognitive function (Yerkes and Dodson 1908; Zaretsky et al. 2024).

### A predictive coding account for cyberdelics

The Predictive Coding (PP) (Friston 2010) framework can be used to understand hallucinatory experience effects. It posits that the brain functions as a predictive machine, continuously generating predictions about sensory inputs balancing prior beliefs and incoming information (Greco et al. 2024). Starting from this premises, The Relaxed Beliefs Under pSychosis (REBUS) model (Carhart-Harris and Friston 2019) suggests that psychedelics induce a relaxation of rigid priors, where this relaxation is primarily mediated by serotonin receptors (5-HT2A receptors) (Lebedev et al. 2015). Such an imbalance between what is expected and what is coming leads then to hallucinations. For instance, Lebedev and colleagues (Lebedev et al. 2015) highlight that under the influence of psilocybin, individuals experience ego dissolution, which can be interpreted through the REBUS framework as a relaxation of the rigid beliefs that normally constrain perception, allowing for a more fluid integration of sensory information. Considering the evidence suggesting similarities between simulated and substance-induced hallucinations on perception, emotional responses, and CF, it might be that cyberdelics have a similar effect. That is, the unexpected nature of the HVVEs might lead to a temporary recalibration of the precision weighting process so that sensory data are considered more reliable than priors. Being sensitive to contextual cues might then make one more open to considering different interpretations. Then, cyberdelics may facilitate a form of cognitive relaxation that aligns with the principles of the REBUS model.

If in the context of substance use such a change is directly linked to serotonin receptors modulation, VR might offer a non-invasive alternative that may modulate serotonin levels indirectly through psychological and physiological mechanisms.

Notably, this perspective could also explain the contrasting findings regarding autonomic responses in this study: facing the prediction error might indeed increase the arousal level, despite the subjective cognitive experience of absorption and relaxation.

### Limitations and future perspectives

Despite its strengths and innovation, this study presents some limitations.

First, the sample consisted exclusively of young adults, limiting the generalisability of the findings. Future research should include diverse samples to explore whether the effects of HVVEs vary across different age groups, as cognitive flexibility, brain connectivity, and autonomic responses are known to change with age (Mevel et al. 2013). Furthermore, certain populations with different psychological profiles (such as anxiety disorders, depression and MCI) might derive greater benefit from HVVEs due to their potential to enhance CF (Dawood Hristova and Pérez-Jover 2023; Magni et al. 2023).

Second, as regards the sample size, while this study is powered to detect medium effects, future studies with larger sample sizes could explore more subtle or smaller effects that may have been beyond the sensitivity of the current design.

Third, we focused on limited autonomic responses: future research should include more specific autonomic measures—e.g., galvanic skin response, blood pressure—to help clarify the relationship between autonomic nervous system activity and the emotional or cognitive shifts facilitated by HVVEs by providing objective data on physiological states, such as emotional arousal, stress reduction, and cognitive engagement, and how these correlate with improved CF.

Additionally, with respect to the measure of CF, future studies could include additional behavioural tasks to address, for example, specific CF subcomponents, such as task switching, set shifting, or problem-solving, providing deeper insights into how HVVEs influence various facets of CF. Moreover, HRV indices were extracted post-task, potentially serving as a stress test, with the initial recording used as a baseline for all conditions. Future studies should implement separate baseline measures for each condition to improve accuracy in assessing HRV changes.

Finally, this study would also benefit from additional control conditions, such as different technologies and videos. This would clarify whether the effects were due to the immersive technology, the content being viewed, or the hallucinatory experience.

## Conclusions

In conclusion, our study provides initial evidence that Cyberdelics can induce measurable changes in CF, emotional states, and autonomic activity. These findings suggest that they be a complementary approach to improving treatments (Carhart-Harris and Nutt 2017), enabling the exploitation of psychedelic advantages without side effects.

## Supplementary Material

Cyberdelics_Supplementary_materials_R1 (Clean).docx

## Data Availability

Data from this study are published in restricted mode on Zenodo repository **(10.5281/zenodo.15286801).** Please contact the corresponding author to access the dataset.
