# Supplementary material for: Cyberdelics: Virtual reality hallucinations modulate cognitive-affective processes
Source: Dialogues Clin Neurosci. 2025 May 23;27(1):1–12. doi: 10.1080/19585969.2025.2499459 (PMC12107671; doi:10.1080/19585969.2025.2499459)
Supplement: Cyberdelics_Supplementary_materials_R1 (Clean).docx [file TDCN_A_2499459_SM6695.docx]

**Supplementary Materials**

**Materials**

**HVVE**

HVVE was developed using DeepDream (DD). DD is a computational method utilized for modifying images, based on a pre-trained deep convolutional neural network (CNN). The process begins by feeding an input image (I) with specific dimensions (width w and height h) through the CNN until a designated layer (Al) is reached. DeepDream modifies the input image by integrating the partial derivatives (gradients) of L regarding the input image. In this research, a higher-level layer (inception_4d/pool) of the GoogleNet CNN was chosen, with hyperparameters set akin to those of the Hallucination Machine (octaves=3, octave scale=1.8, iterations=16, jitter=32, zoom=1, step size=1.5, flow threshold=6, blending ratio for optical flow=0.9, blending ratio for background=0.1; Rastelli et al., 2020).

**Alternative Use Test (AUT)**

The AUT responses were evaluated according to the traditional approach, proposed by Vartanin and colleagues (2020). Each participant's responses were scored for (1) fluency (number of ideas generated), (2) flexibility (number of different conceptual categories, especially pleasure, utility, art, aggressiveness; present = 1, everything else = 0), (3) originality (unique responses; single = 1, all else = 0). Average score values were calculated to obtain a total score of the flexibility and fluency subscales, while for originality the values (0 and 1) were summed.

**Autonomic Measures - Descriptions**

The table reports abbreviations' meanings and autonomic measures explanation.

| *Abbreviation* | *Meaning* | *Domain* | *Description* |
| --- | --- | --- | --- |
|  |  |  |  |
| **HR** | Heart Rate | Time | The pulse rate measures the number of heartbeats per minute (bpm). |
| **Mean RR** | Mean RR | Time | Mean of interval RR, namely time interval between heartbeats. |
|  |  |  |  |
| **SDNN** | Standard deviation of NN intervals | Time | It quantifies the overall variation in time between consecutive normal heartbeats. |
|  |  |  |  |
| **RMSSD** | Root mean square of successive RR interval differences | Time | It quantifies the short-term variations in the heart rate by examining the differences between consecutive RR intervals. |
| **LF** | Low Frequency Power | Frequency | Absolute power of the low-frequency band (0.04–0.15 Hz). LF is associated with sympathetic activity (stress response). |
|  |  |  |  |
| **HF** | High Frequency Power | Frequency | Absolute power of the high-frequency band (0.15–0.4 Hz). HF is associated with parasympathetic activity (relaxation). |
|  |  |  |  |
| **LF/HF Ratio** | Ratio between Low and High Frequency Power | Frequency | A higher low-frequency to high-frequency ratio often indicates higher stress levels. |
|  |  |  |  |
| **SI** | Stress Index | Composite Index | Parameter that indicates the degree of sympathetic nervous system (SNS) dominance over the parasympathetic nervous system (PNS). |
|  |  |  |  |
| **RR** | Respiratory Rate | Composite Index | This indicates the rate of breathing (number of breaths per minute). |
|  |  |  |  |
| **PNS** | Parasympathetic Nervous System | Composite Index | Index reflecting parasympathetic autonomic activity. |
|  |  |  |  |
| **SNS** | Sympathetic Nervous System | Composite Index | Index reflecting sympathetic autonomic activity. |

**Variables of interest - descriptives statistics**

|  | **Baseline** | | **HVVE** | | **CC** | |
| --- | --- | --- | --- | --- | --- | --- |
|  | ***M (SD)*** | ***(Min-max)*** | ***M (SD)*** | ***(Min-max)*** | ***M (SD)*** | ***(Min-max)*** |
| *Cognitive Flexibility* | | | | | | |
| **AUT** |  |  |  |  |  |  |
| Flexibility | 0.44 (0.12) | 0.25-0.63 | 0.53 (0.24) | 0.00-1.75 | 0.42 (0.14) | 0.00-0.63 |
| Originality | 1.42 (0.67) | 0.00-2.00 | 1.53 (0.64) | 0.00-2.00 | 1.53 (0.76) | 0.00-2.00 |
| Fluency | 6.19 (2.85) | 1.00-14.50 | 6.22 (3.99) | 0.00-19.00 | 5.59 (3.54) | 0.00-18.00 |
| **SCW** |  |  |  |  |  |  |
| STIR | 0.45 (0.31) | 2.90×10-4 - 1.32 | 0.31 (0.32) | 0.02 – 1.46 | 0.49 (0.33) | 0.03 – 2.14 |
| *Anxiety and Affective States* | | | | | | |
| **FSS** |  |  |  |  |  |  |
| Flow |  |  | 42.34 (8.61) | 25-61 | 44.02 (10.30) | 26-60 |
| Worry |  |  | 8.50 (5.13) | 3-20 | 8.06 (5.21) | 3-21 |
| Fluency |  |  | 25.28 (6.17) | 14-41 | 28.42 (4.77) | 14-42 |
| Absorption |  |  | 17.06 (3.97) | 8-26 | 15.60 (4.77) | 6-25 |
|  |  |  |  |  |  |  |
| **STAI-S** | 48.52 (2.83) | 42-54 | 38.62 (7.80) | 23-63 | 32.92 (7.12) | 21-56 |
|  |  |  |  |  |  |  |
| **PANAS** |  |  |  |  |  |  |
| Positive | 28.24 (7.01) | 16-45 | 28.24 (7.01) | 16-45 | 27.62 (7.26) | 10-42 |
| Negative | 17.42 (6.12) | 10-33 | 14.36 (4.59) | 10-311 | 1.86 (2.63) | 10-21 |
|  |  |  |  |  |  |  |
| **SAM** |  |  |  |  |  |  |
| Valence | 3.78 (0.54) | 3-5 | 3.60 (0.80) | 2-5 | 3.90 (0.73) | 2-5 |
| Arousal | 2.90 (0.95) | 1-5 | 3.00 (1.10) | 1-5 | 2.32 (1.22) | 1-5 |
| Dominance | 2.78 (0.91) | 1-5 | 3.00 (0.96) | 1-5 | 2.84 (1.05) | 1-5 |
|  |  |  |  |  |  |  |
| *Autonomic measures* | | | | | | |
| **HR** | 82.48 (13.08) | 50 - 112 | 78.34 (11.85) | 46-100 | 78.69 (10.50) | 60-101 |
| **Mean RR** | 736.77 (109.50) | 536.22-997.45 | 772.70 (107.58) | 601.45-1040.28 | 775.92 (101.70) | 595.08-994.97 |
| **SDNN** | 47.91 (22.84) | 13.82-106.54 | 46.42 (16.68) | 13.64-88.95 | 49.57 (17.51) | 18.26-91.49 |
| **LF/HF Ratio** | 2.02 (1.60) | 0.14 – 6.44 | 2.19 (1.73) | 0.33-6.44 | 2.45 (1.92) | 0.32-6.06 |
| **Respiratory Rate** | 13.83 (3.50) | 6.27-20.86 | 14.19 (3.63) | 8.11-22.24 | 13.92 (3.62) | 5.60-21.54 |
| **PNS** | -0.89 (1.03) | -2.71-1.65 | -0.80 (0.82) | -2.19-1.37 | -0.52 (1.01) | -2.15-2.10 |
| **SNS** | 1.44 (1.52) | -1.89-5.62 | 1.04 (1.10) | -0.88-3.01 | 0.83 (1.16) | -1.99-3.47 |
| **SI** | 11.49 (4.94) | 2.84-24.51 | 10.44 (3.76) | 3.95-20.15 | 9.77 (3.99) | 2.87-25.87 |
| **NL RMSSD** | 3.51 (0.49) | 2.30-4.45 | 3.56 (0.38) | 2.70-4.36 | 3.67 (0.42) | 2.77-4.41 |
| **NL LF** | 6.61 (1.15) | 4.36-8.58 | 6.71 (1.09) | 3.38-8.33 | 6.89 (0.90) | 4.54-9.13 |
| **NL HF** | 6.09 (1.03) | 3.06-7.74 | 6.04 (0.73) | 3.06-7.74 | 6.37 (0.77) | 4.22-7.56 |

**Anova Results**

This table presents the results of the Repeated measures ANOVA for a within-subjects design.; MS = Mean Square; F = F-statistic, df= degrees of freedom, η²_p_ = eta square. *****=0.05, ******=0.01, *******= <0.001.

|  | ***MS*** | ***F*** | ***df*** | ***P-value*** | ***η²_p_*** |
| --- | --- | --- | --- | --- | --- |
| *Anxiety and Affective States* | | | | | |
| **STAI-S** | 3115.50 (31.94) | 97.53 | 2, 98 | <.001*** | 0.66 |
| **FSS**  Flow  Absorption  Fluency | 70.56 (36.90)  53.29 (10.27)  246.49 (19.18) | 1.91  5.18  12.84 | 1, 49  1, 49  1, 49 | 0.17  0.027**  <.001*** | 0.03  0.09  0.20 |
| **PANAS**  Positive | 130.84 (14.65) | 8.92 | 2, 98 | <.001*** | 0.15 |
| **SAM**  Valence  Arousal  Dominance | 1.14 (0.39)  13.48 (57.85)  0.77 (0.88) | 2.86  11.41  0.87 | 2, 98  1.75, 0.67  1.66, 81.77 | 0.06  <.001***  0.40 | 0.05  0.18  0.01 |
| *Autonomic measures* | | | | | |
| **HR** | 349.46 (31.33) | 11.15 | 1.60, 70.45 | <.001*** | 0.20 |
| **Mean RR** | 21730.30 (1910.26) | 11.37 | 2, 90 | <.001*** | 0.20 |
| **SDNN** | 186.51 (210.32) | 0.88 | 1.62, 61.87 | 0.39 | 0.02 |
| **Respiratory Rate** | 1.28 (9.01) | 0.14 | 2, 92 | 0.867 | 0.003 |
| **PNS index** | 1.22 (0.28) | 4.30 | 2, 84 | 0.017** | 0.09 |
| **SNS index** | 5.95 (0.59) | 9.94 | 1.48, 62.29 | <.001*** | 0.19 |
| **SI** | 50.63 (8.86) | 5.77 | 1.54, 66.28 | 0.009** | 0.11 |
| **NL RMSSD** | 0.33 (0.09) | 3.64 | 1.54, 60.37 | 0.043** | 0.08 |

This table presents the results of the post hoc Bonferroni tests following a RM ANOVA for a within-subjects design. B= baseline; CC= Control Condition; HVVE= HVVE Condition; MD = Mean Difference; SE = Standard error; t = t-statistic for each comparison; Cohen’s d = Cohen’s d effect size; p-value (adj) = Bonferroni-adjusted p-value for each comparison. *****=0.05, ******=0.01, *******= <0.001.

|  | ***Comparison*** | ***MD [95% CI]*** | ***SE*** | ***t*** | ***Cohen’s d [95% CI]*** | ***P-value (adj)*** |
| --- | --- | --- | --- | --- | --- | --- |
| *Anxiety and Affective States* | | | | | | |
| **STAI-S** | B - HVVE  B – CC  HVVE – CC | 9.90 [7.14, 12.65]  15.60 [12.84, 18.35]  5.70 [2.94, 8.45] | 1.13  1.13  1.13 | 8.75  13.80  5.04 | 1.56 [0.98, 2.14]  2.46 [1.72, 3.21]  0.90 [0.41, 1.38] | < .001***  < .001***  < .001*** |
| **PANAS**  Positive | B – HVVE  B – CC  HVVE – CC | 2.44 [0.57, 4.30]  3.06 [1.19, 4.92]  0.62 [-1.24, 2.48] | 0.76  0.76  0.76 | 3.18  3.99  0.81 | 0.37 [0.07, 0.66]  0.46 [0.15, 0.77]  0.09 [-0.19, 0.37] | .006**  < .001***  1.00 |
|  |  |  |  |  |  |  |
| **SAM**  Arousal | B - HVVE  B – CC  HVVE - CC | -0.10 [-0.47, 0.27]  0.58 [0.20, 0.95]  0.68 [0.30, 1.05] | 0.15  0.15  0.15 | -0.65  3.74  4.42 | -0.09 [-0.43, 0.25]  0.52 [0.16, 0.89]  0.61 [0.24, 0.99] | 1.00  < .001***  < .001*** |
| *Autonomic measures* | | | | | | |
| **HR** | B – HVVE  B – CC  HVVE - CC | 4.06 [1.49, 6.64]  4.53 [1.95, 7.11]  0.46 [-2.11, 3.04] | 1.49  1.95  -2.11 | 3.85  4.29  0.44 | 0.35 [0.11, 0.60]  0.40 [0.14, 0.65]  0.04 [-0.18, 0.27] | < .001***  < .001***  1.00 |
| **PNS index** | B – HVVE  B – CC  HVVE – CC | -0.12 [-0.40, 0.16]  -0.33 [-0.33, -0.61]  -0.21 [-0.49, 0.07] | 0.11  0.11  0.11 | -1.06  -2.89  -1.83 | -0.13 [-0.43, 0.17]  -0.35 [-0.67, -0.04]  -0.22 [-0.53, 0.08] | 0.87  .014**  0.21 |
| **SNS index** | B – HVVE  B – CC  HVVE - CC | 0.45 [0.10, 0.80]  0.62 [0.27, 0.97]  0.16 [-0.18, 0.52] | 0.14  0.14  0.14 | 3.14  4.31  1.17 | 0.37 [0.06, 0.68]  0.51 [0.19, 0.84]  0.14 [-0.15, 0.43] | 0.007**  < .001***  0.73 |
| **SI** | B – HVVE  B – CC  HVVE - CC | 1.09 [-0.27, 2.45]  1.87 [0.51, 3.23]  0.78 [-0.57, 2.14] | 0.55  0.55  0.55 | 1.95  3.36  1.40 | 0.27 [-0,07, 0.62]  0.47 [0.10, 0.83]  0.19 [-0.15, 0.54] | 0.16  0.003**  0.48 |
| **NL RMSSD** | B – HVVE  B – CC  HVVE - CC | -0.07 [-0.22, 0.06]  -0.16 [-0.30, -0.01]  -0.08 [-0.22, 0.06] | 0.05  0.05  0.05 | -1.32  -2.69  -1.37 | -0.18 [-0.54, 0.16]  -0.38 [-0.75, -0.01]  -0.19 [-0.55, 0.15] | 0.57  0.026**  0.51 |

This table presents the results of the Friedman test for a within-subjects design. Chi-Squared= Chi Squared Test; Kendall’s W= Kendall's Coefficient of Concordance. *****=0.05, ******=0.01, *******= <0.001.

|  | **Kendall’s W** | **Chi-Squared** | **P-value** |
| --- | --- | --- | --- |
| *Cognitive Flexibility* | | | |
| **AUT**  Flexibility  Originality  Fluency | 0.140  0.034  0.038 | 13.73  3.31  3.74 | 0.001***  0.19  0.15 |
| **SCW**  STIR | 0.11 | 11.08 | 0.004** |
| *Anxiety and Affective States* | | | |
| **SUDS** | 0.19 | 19.16 | < .001*** |
|  |  |  |  |
| **PANAS**  Negative | 0.35 | 35.00 | <.001*** |
| **FSS**  Worry | 0.008 | 0.40 | 0.52 |
| *Autonomic measures* | | | |
| **LF/HF ratio** | 0.03 | 3.31 | 0.19 |
| **NL LF** | 0.03 | 2.22 | 0.32 |
| **NL HF** | 0.04 | 3.21 | 0.20 |

This table presents the results of the post Conover’s test following a Friedman test for a within-subjects design. B= baseline; CC= Control Condition; HVVE= Experimental Condition; t = t-statistic for each comparison; p-value (adj) = Bonferroni-adjusted p-value for each comparison. *****=0.05, ******=0.01, *******= <0.001.

|  | ***Comparison*** | ***t*** | ***P-value (adj)*** |
| --- | --- | --- | --- |
| *Cognitive flexibility* | | | |
| **AUT**  Flexibility | B - HVVE  B – CC  HVVE – CC | 2.68  0.89  3.57 | 0.02**  1.000  0.002** |
|  |  |  |  |
| **SCW**  STIR | B - HVVE  B – CC  HVVE – CC | 2.60  0.50  3.10 | 0.03**  1.00  0.008** |
|  |  |  |  |
| *Anxiety and Affective States* | | | |
| **SUDS** | B - HVVE  B – CC  HVVE – CC | 1.73  2.63  4.36 | 0.25  0.030**  < .001*** |
|  |  |  |  |
| **PANAS**  Negative | B - HVVE  B – CC  HVVE – CC | 1.95  5.82  3.86 | 0.15  < .001***  < .001*** |

Results showed a significant main effect of Condition (F(2, 84) = 4.30, p = 0.01, η²p = 0.09) for the PNS Index. Post hoc comparisons revealed that CC (M= -0.60, SD= 0.98) significantly reduced PNS activity compared to Baseline (M = -0.93, SD = 1.00) (p = .014). The Baseline and HHVE (M= -0.81, SD = 0.82) comparison did not show a significant mean difference as the HVVE and CC comparison.

Results showed a significant main effect of Condition (F(1.54, 66.28) = 5.71, p = 0.009, η²p = 0.11) for SI. Post hoc comparisons revealed that CC (M = 9.40, SD = 3.18) significantly reduced SI compared to Baseline (M = 11.28, SD = 4.92) (p = .003). The Baseline and HHVE (M= 10.19, SD = 3.59) comparison did not show a significant mean difference as the HVVE and CC comparison.

Results showed a significant main effect of Condition (F(1.54, 60.37) = 3.64, p = 0.04, η²p = 0.08) for NL RMSSD. Post hoc comparisons revealed that CC (M= 3.61, SD = 0.39) significantly increased NL RMSSD compared to Baseline (M = 3.45, SD = 0.47) (p = .026). The Baseline and HHVE (M= 3.53, SD = 0.37) comparison did not show a significant mean difference as the HVVE and CC comparison.
